# International Framework for Cancer Patient Advocacy: Empowering Organizations and Patients to Create a National Call to Action on Cancer

**DOI:** 10.1200/JGO.2015.000398

**Published:** 2015-10-28

**Authors:** Rebekkah M. Schear, Leigh Manasco, Devon McGoldrick, Kiti Kajana, Lauren Rosenthal, Ann McMikel, Nancy Lins

**Affiliations:** Rebekkah M. Schear and Devon McGoldrick, LIVESTRONG Foundation, Austin, TX; Leigh Manasco, LIVESTRONG Foundation, Memphis, TN; Kiti Kajana, Open Society Foundations, New York, NY; Lauren Rosenthal and Ann McMikel, American Cancer Society; and Nancy Lins, N.E. Lins & Associates, Atlanta, GA.

## Abstract

**Purpose:**

With the rate of cancer and other noncommunicable diseases (NCDs) growing globally, cancer prevention and control efforts are critical internationally. Moreover, since the 2011 United Nations High-Level Meeting on NCDs, the international health and development community has shifted its awareness to include NCDs as a global health priority, especially in developing countries where mortality rates are disproportionately high. Simultaneously, with the dissemination of the World Cancer Declaration and the evolution of cancer control policies, the international cancer community has recognized the value of engaging patients in reducing the global cancer burden. Cancer advocacy programs that involve patients, survivors, and nongovernmental organizations (NGOs) have increasing opportunities for global impact.

**Methods:**

We developed a framework over 4 years through implementation of two pilot projects. We created a series of trainings and tools to build the capacity of local NGOs and patients to plan and implement a forum for patients with cancer and to create and disseminate a national call to action. The framework was piloted in South Africa from 2009 to 2011 and Japan from 2012 to 2014, and results were measured through postproject surveys completed by members of the collaborative working group and interviews with the in-country partner.

**Results:**

The framework is globally relevant and could be adapted and implemented in low- and middle-income countries to amplify patient voices in the policymaking process, increase grassroots mobilization, and improve health systems and infrastructure through addressing patient needs.

**Conclusion:**

With the dominant paradigm of global health in developing countries—which has previously focused on HIV/AIDS, maternal and child health, tuberculosis, and malaria—shifting to adapt to the burgeoning NCD burden, effective patient-centered advocacy frameworks are critical to the success of NCD control.

## INTRODUCTION

Cancer is a leading cause of death around the world.^1^ The global cancer community has acknowledged that without significant efforts, rates of new cancer cases could rise from 14 to 22 million annually in the next two decades.^1^ Advocacy plays a pivotal role in cancer control planning^2,3^ and is necessary in any resource setting to influence policy and improve delivery of cancer control.^4^ The WHO 2008 practical guide on cancer control policy and advocacy and 2013 to 2020 Global Action Plan for the Prevention and Control of Noncommunicable Diseases (NCDs) both articulate that advocacy is a critical strategy in improving cancer control and care delivery nationally and globally.^5,6^

Patients with cancer, survivors, and caregivers are in an ideal position to provide insight into gaps within systems of care. Through capacity building, their voices are a powerful tool in advocating for improvements in knowledge, practice, policy, and services and in empowering others to share their stories. Moreover, cancer patient advocacy supports national efforts toward achievement of NCD and cancer control targets, such as those outlined in the Global Action Plan and in the World Cancer Declaration. This article presents a framework for empowering patients, survivors, and organizations to implement a comprehensive, patient-informed national call to action on cancer through planning, implementing, and leveraging outcomes of a patient forum.

## CONCEPT OF CANCER PATIENT ADVOCACY

Definitions of cancer patient advocacy vary in the literature; here, we define this advocacy as a systematic approach to promoting a cancer-related issue and motivating others to take action. This includes policy advocacy through both grasstops and grassroots approaches.^7^ Cancer patient advocacy provides a framework to ensure meaningful involvement of the community in decisions affecting patient lives.^8^ It is recognized that cancer patient advocacy provides a voice for patients and raises awareness of needs^8,9^; patient advocacy is also deeply rooted in the concept of empowerment, which, according to the WHO and Gray and Doan,^10,11^ posits that individuals understand their own needs better than others and that it is ideal for people to have control in shaping the direction and events of their lives.^11,12^ Furthermore, the concept of participation builds on the idea of empowerment, because empowering individuals implies providing for active participation in their own care.^12^

## ORIGINS AND THE GROWING GLOBAL MOVEMENT

The history of cancer patient advocacy varies globally. In the West, it has had a robust history over the last 30 years. In the United States in particular, it has roots in the successful civil, women's, human, and consumer rights and HIV/AIDS advocacy movements.^13–16^ Pioneering organizations of cancer advocacy, such as American Cancer Society, began implementing patient engagement activities as early as the 1950s (with Reach to Recovery program), and others like Susan G. Komen for the Cure and the National Coalition for Cancer Survivorship launched major activities in the 1980s and 1990s. Rapid growth of these activities, particularly among breast cancer organizations, successfully led to policy changes to benefit survivors, improvement in awareness about survivorship, and the introduction of survivor-driven research.^2,14,16–18^ Over the last few decades, cancer advocacy in the United States has led to advocate groups gaining significant influence over policymakers, researchers, and health care providers^19^ and has become a critical part of the shift in the health care paradigm from illness-centered to patient-centered care, where patients and families are more actively participating in their care and in the creation of services and policies.^19–21^

Although organizations in countries such as Indonesia, Egypt, Canada, Chile, and the United Kingdom have engaged in cancer advocacy for more than 15 years,^4,19,22^ in many other countries, cancer advocacy is a more novel concept. The ethos and language of advocacy lack consistency across many cultures, and the word for advocacy is often not translatable directly.^15,18^ That said, there is current growth in advocacy activities in many low- and middle-income countries (LMICs), particularly in Africa and Latin America.^23–25^ This burgeoning activity is evident in the context of the massive call to action issued through the 2011 United Nations Summit on NCDs and the resulting Global Action Plan, Monitoring Framework, and Global Coordination Mechanism, which have evolved as part of the large-scale response to NCD and cancer problems.^26^

The WHO reported that as of 2010, 107 countries had national operational policies or action plans for cancer.^27^ However, the reality at the local level, especially in LMICs, is that there remains a dearth of infrastructure to address cancer,^28,29^ and it is likely that physical, emotional, and practical needs of patients are not being fully met. Patient advocacy can improve access to treatment, raise awareness of the value of prevention,^19,30^ and ensure that patient viewpoints are integrated into planning and policy.^15^

## PATIENT FORUMS

Cancer patient forums are stakeholder meetings addressing a broad range of quality-of-life and survivorship issues. The objectives of forums are determined by local stakeholders but may include providing data, raising awareness of key system issues from the patient perspective, or improving resources and infrastructure.^31^ Forums convene dynamic groups of stakeholders like nongovernmental organization (NGO) leaders, patients, survivors, caregivers, health care professionals, media, government leaders, and researchers to dialogue.^31^

The implementation of structured patient forums was undertaken by the Union for International Cancer Control (UICC) in 2004, as a result of reports of similar successful initiatives in France and Italy in the late 1990s, and was based on the impact of the US President's Cancer Panels. We built on this initial concept by expanding the best practices of the UICC and creating a structured, replicable framework for patient forum implementation.

The International Patient Advocacy Framework (IPAF) was developed to support a comprehensive, patient-informed national call to action on cancer through planning, implementing, and leveraging the outcomes of patient forums. It proposed elevating patient and survivor voices to bring visibility to gaps in cancer control and highlight the need for cancer to be a higher priority on national health agendas.

Long-term outcomes of implementation of the IPAF are two-fold: 1) contribution toward addressing needs of patients with cancer nationally through improvements in attitudes, knowledge, practice, policy, systems, and services; and 2) national development of a new or existing patient advocacy movement.

## METHODS

**Figure 1:**
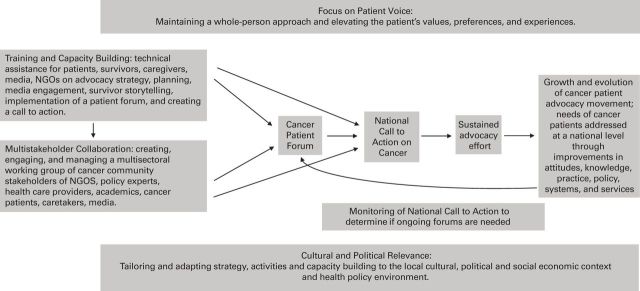
Components of conceptual framework. Policy environment can change from highly restrictive to receptive,^32^ and throughout a long-term advocacy campaign, varying strategies will be used to adapt to the evolving political environment.^32^ It is therefore critical to assess environment in advance of planning advocacy effort to maximize potential impact.

Figure 1 presents the IPAF. The framework was developed over 4 years through implementation of two pilot projects in different countries. Using the program evaluation of the UICC, we created a series of trainings and tools to build the capacity of local NGOs, patients, and survivors to plan and implement a patient forum and to create and disseminate a national call to action. Pilot countries were selected based on criteria including: substantial cancer incidence and survivorship rates, some history of advocacy, basic level of care infrastructure, base of cancer civil society organizations, and strong media infrastructure. South Africa and Japan were selected with the intent of testing the framework in different contexts.

The framework was piloted in South Africa from 2009 to 2011, and results were measured through postproject surveys completed by members of the collaborative working group and interviews with the in-country partner (lead agency). Results from surveys were analyzed, and minor changes were made to the framework. It was then piloted in Japan from 2012 to 2014. Results were measured again in the same means.

In both countries, the first step in launching the project was to select a lead agency with responsibility for building a working group to support planning and implementation of the forum, launching a call to action, acting as secretariat for the working group, facilitating administrative duties, and guiding strategic vision for the long-term advocacy effort. A seed grant was awarded to the lead agency to support forum preparation. An initial strategy session was held, which resulted in a logic model and project plan.

## APPLICATION OF FRAMEWORK

Table 1 lists key aspects of the logic model for both pilots implementing the framework. As Clark and Stovall^11^ note, there is a necessary process to transition from a cancer survivor to an advocate, which “requires development of new skills, such as information seeking, communication, problem solving, and negotiation.”^11p240^ For patients and NGOs to be effective change agents, it is critical that they possess the competences to communicate with researchers and providers^14^ as well as policymakers and media. The framework incorporates in-person trainings on topics including:

- Grasstops advocacy fundamentals
- Identifying and prioritizing issues and solutions
- Crafting and delivering messages
- Creating and launching a national call to action
- Action planning (grassroots campaign and patient forum)

**Table 1:** Logic Model for Pilot Projects in South Africa and Japan

Factor	Description
Goals	Empower patients to engage in policy making process; raise awareness among policymakers, general public, health care providers, and media about challenges faced by patients with cancer
Objectives	Implement cancer patient forums with planning committee of NGOs that provides data and information about cancer, raises awareness of select cancer issues, and provides case for improving resources and infrastructure to address cancer issues and break down social stigmas associated with cancer
Timeframe	18 months
Inputs	Training materials, human capital, funding, office infrastructure and supplies
Activities	Training and education, patient forum, national call to action, stakeholder collaboration, patient mobilization
Outputs	Stakeholder meeting held; two to three trainings held; patient forum hosted; national call to action launched and disseminated; media campaign implemented
Potential short-term outcomes	Trained patients empowered to share their stories; media trained to include patient voice; increase in knowledge of general public about patient experiences; improved relationships between journalists and NGOs and journalists and patients; national call to action reaches cancer control policymakers; patient advocates engage with key policymakers
Potential intermediate-term outcomes	Increase in media coverage and exposure for patient stories; increased collaboration among NGOs; increase in corporate engagement in advocacy efforts
Potential long-term outcomes	Needs of patients with cancer addressed at national level through improvements in attitudes, knowledge, practice, policy, systems, and services; increased social support and reduced social stigma and discrimination for people affected by cancer

Abbreviation: NGO, nongovernmental organization.

In the 12 months after initial trainings, ongoing technical assistance was provided, and the local working group produced outputs including but not limited to: vision for the goals and objectives of the forum, agenda, fundraising strategy, community engagement activities, dissemination plan for the call to action (including social media strategy), and a monitoring and evaluation plan. Additional in-person media trainings were also conducted before the forum launch.

## RESULTS

Table 2 lists the outcomes of both pilot projects. It is acknowledged that evaluating advocacy is inherently challenging and requires a different approach than evaluation of programs, given the nonlinear nature of advocacy efforts and the rapidly changing environment in which they occur.^32–34^ In addition, best practices in advocacy evaluation articulate that measurement of intermediate outcomes, such as capacity building, is just as critical as assessment of a final outcome, such as policy change.^32–35^ Given this and the fact that long-term results in both countries are still being assessed, our results focus mostly on short- and intermediate-term impacts.

**Table 2:** Results of Implementation of IPAF in South Africa and Japan

Goals of Framework	Results	
	South Africa	Japan
Short-term outcomes		
Trained patients empowered and prepared to share their stories at forum	11 patients trained and shared stories at forum	30 patients trained; nine shared stories at forum
NGO working group prepared to implement patient forum	Patient forum held	Patient forum held
	83% of working group members (n = 6) said training and capacity building tools of framework “helped a lot” in planning and implementation of forum	100% of working group members (n = 5) said training and capacity building tools of framework were “very useful” or “useful” for planning and implementation of forum
Creation and release of call to action on cancer	Call to action on cancer written and released	Call to action on cancer written and released
Intermediate-term outcomes		
Increase in attention for cancer issues and patient voices	> 34 articles and interviews published about survivor stories and call to action after its release	Base of trained patient advocates actively sought to represent patient perspective in media (via television, print); increased inclusion of patient stories and panels in academic settings (eg, conferences like Annual Meeting of Japan Lung Cancer Society)
Increased collaboration; new partnerships	Formation of first cancer alliance in country by NGOs and patient representatives	New partnerships formed between working group and other NGOs; improved relationship between cancer support organizations (increase of collaboration through organization of joint events and increase of positive communications)
Participation of civil society in policy discussions with decisionmakers and/or growth of patient-driven grassroots movement	Cancer alliance invited to join and actively participating in Ministerial Advisory Committee on Cancer	Increased public engagement (ongoing social media interactions with public through OCT campaign social media mechanisms)
	Cancer survivors invited to formally partner with government and are sharing stories with local communities in cancer awareness efforts	Additional survivor speaking trainings facilitated by coalition of NGOs after pilot conclusion to sustain base of trained survivor advocates who can share their stories
Long-term outcomes		
Needs of patients with cancer addressed at national level through improvements in attitudes, knowledge, practice, policy, systems, services	Development of new national cancer control policies; government commitment to develop national breast cancer policy and ensure national cancer registry is mobilized and adequately resourced	Still determining

Abbreviations: IPAF, International Patient Advocacy Framework; NGO, nongovernmental organization; OCT, Over Cancer Together.

Results illustrate that the framework empowered organizations and patients to create and launch a national call to action on cancer through implementing a patient forum. Although the sharing of patient voices can happen in a variety of settings, the structure of the framework as an advocacy tool makes it a unique mechanism for patients to share their experiences in a setting that ultimately can contribute toward addressing patient needs through increasing community awareness and mobilization, improving services and resources, and changing policy over time. Involving patients and caregivers in the advocacy process brings a human face and a compelling urgency to addressing cancer, and integrating the voices of marginalized populations helps to ensure that cancer control strategies are person centered.^4^ These results build on the small-wins approach, which posits that successes in advocacy that unfold in an incremental fashion are needed because they “can set in motion forces that can lead to increased higher level interventions”^8p313^; the results we saw in South Africa and Japan are small wins and are indicative that with continued advocacy activity, greater impact is a possible trajectory.

There are several examples of similar models of cancer advocacy implemented in the United States over the last decade illustrating longer term impact. Two examples are summarized in Table 3. Models like those of the Adolescent and Young Adult Oncology Progress Review Group and the National Action Plan for Cancer Survivorship, which employed frameworks similar to that of the IPAF through multistakeholder collaboration and inclusion of patient voices, present compelling evidence to demonstrate the potential of this type of patient advocacy approach for program and policy development and improvement in cancer care and control.

**Table 3:** Examples of Long-Term Impact of Similar Advocacy Initiatives

Initiative	NAPCS	AYAO PRG
Involved organizations	Centers for Disease Control, LIVESTRONG Foundation	National Cancer Institute
Year of initiation	2004	2005
Summary	Plan resulted from deliberation of panel of survivors, clinicians, and researchers; included 28 priority public health needs of cancer survivors and 96 strategies to address these needs; objective was to provide “guide to national, state, and local public organizations to address the needs of survivors and allocate resources to cancer survivorship initiatives”^35ap426^	Composed of diverse cross section of experts and survivors to identify priorities for improving outcomes of AYAs diagnosed with cancer; released report with five calls to action,* and 3 months later, coalition of organizations was created to move advocacy agenda forward
Impact results	In follow-up assessment to monitor implementation, 76% of initially recommended strategies had activities implemented in areas of surveillance and applied research; communication, education, and training; and programs, policy, and infrastructure, between 1 and 3 years after launch^35a,36^	No. of retrospective analyses provided evidence for biologic distinctiveness of some cancers in AYAs; unique characteristics of AYAs identified; professional education programs developed and implemented (eg, American Society of Clinical Oncology and Nurse Oncology Education Program); national Young Adult Cancer Awareness Week exists; AYA-specific committees are part of National Clinical Trials Network; national AYA guidelines created; International Charter of Rights for Young People With Cancer developed and promoted; increase in published papers on subject, from < 300 before PRG to > 5,000 in 2010,^37^ showing that this model launched new field of study and growing grassroots movement

Abbreviations: AYA, adolescent and young adult; AYAO PRG, Adolescent and Young Adult Oncology Progress Review Group; NAPCS, National Action Plan for Cancer Survivorship.

* Calls to action included identify characteristics that distinguish unique cancer burden in AYA patients; provide education, training, and communication to improve awareness, prevention, access, and quality cancer care for AYAs; create tools to study AYA cancer problem; ensure excellence in service delivery across cancer control continuum (ie, prevention, screening, diagnosis, treatment, survivorship, and end of life); strengthen and promote advocacy and support of AYA patients with cancer.

The South African forum marked the first time to our knowledge that patients, civil society, policymakers, and health care providers gathered to engage in dialogue about cancer control. In the follow-up survey, one member of the working group in South Africa noted that the greatest success of the forum was that “government attended and heard our voices.” The forum and call to action marked important first steps on the road toward policymakers considering patient views in the policymaking process. This is consistent with the idea that “government leaders often recognize the value of responding to the needs of their constituents ... when those needs become highly visible,”^30p352^ and “finding ways of genuinely involving cancer [survivors] will greatly strengthen advocacy in the long run.”^4p20^

In Japan, stakeholders sought to build awareness of the challenges that patients face among the public and create a base of informed community members who could mobilize to support a grassroots campaign. Results such as the ongoing inclusion of patient voices in media postforum and release of the call to action and growth of the related Over Cancer Together campaign indicate early success. These results are echoed in the literature, which notes that a critical aspect of advocacy is strategic communication, which informs community about challenges and mobilizes it to find solutions collaboratively.^4^

The framework supported both country initiatives in achieving their different goals: a grasstops approach in South Africa and a grassroots approach in Japan. We suggest that this occurred as a result of several critical success factors, summarized in Table 4.

**Table 4:** Critical Success Factors for Implementation of IPAF

Factor	Description
Historical precedent and cultural value of advocacy in country*	History of and cultural value for concept; if country has little to no history of advocacy, it may be more challenging to develop understanding of concept and nuances; if advocacy has limited cultural value or is contrary to political structures in country, basic tenets of advocacy strategy may have limited utility, or it may take larger-scale effort to see results
Basic cancer care infrastructure†	Some level of existing cancer control plans and systems; if country has little infrastructure to detect or treat cancer, there are likely lower rates of survivorship, which can pose challenge when building base of advocates
Existence of organizations with basic knowledge of and interest in advocacy	Organizations that are invested in and have some knowledge of cancer advocacy; furthermore, working with organizations that already pursue some type of advocacy activity will increase likelihood of sustainability
Media infrastructure‡	Basic traditional and new media infrastructure; success in building grassroots movement largely depends on leveraging media to bring awareness to patient stories and issues being raised

Abbreviation: IPAF, International Patient Advocacy Framework.

* South Africa in particular has a robust history of HIV/AIDS advocacy.

† Japan in particular has evolved cancer care infrastructure.

‡ Research has established that “successful advocacy includes use of the media to educate the public on key problems and issues as well as the strategies to address these problems … and is thought to play a significant role in shaping public opinion, which in turn may shape the policy agenda.”^38p294^

## DISCUSSION

Patient advocacy can be challenging as a result of stigma, which acts as a significant barrier to accurate surveillance (because of individuals fearing diagnosis or treatment)^39^ and has led to challenges in survivors sharing their cancer experiences for fear of judgment or discrimination. In some cases, the storytelling training can act as a means to reduce stigma; however, stigma and fear may prevent patients and survivors from desiring to participate. That being said, the inclusion of raw, powerful patient stories in both countries was a significant contributor to success of the model because of the transportation effects of storytelling. This supports existing data suggesting that stories can change attitudes and affect beliefs and action “by presenting emotionally powerful information along with vivid mental images, while reducing counter arguing.”^36pS175^

The WHO notes that “coalition building ... and social mobilization are critical to the success of advocacy efforts”^4p19^; in addition, “involving health-care professionals and researchers is also critical as this ensures that advocacy plans are evidence-based and relevant.”^4p7^ Furthermore, experts state that ultimately, the benefits of collaboration in advocacy include “a reduction in redundancy of services, conservation of human and financial resources ... and a population of survivors who are empowered and engaged advocates.”^7p2313^ However, cultivating meaningful collaboration between NGOs and other stakeholders is a challenging and nuanced endeavor as a result of competition (for funding, awareness, and public engagement) and competing priorities. We found that it is critical for partnering NGOs to build trust and find a shared vision of ultimate benefit for all partners, one that surmounts individual organizational goals toward a more powerful common agenda.

There are limitations to our framework. One is the fact that it has only been tested in two countries. We anticipate that additional testing in other regions will be valuable in further demonstrating the impact of the framework as a global model.

As discussed previously, it was determined at the outset of both pilots that we would only measure the short- and intermediate-term impacts. It was requested that lead agencies work collaboratively to track and share long-term impacts, but this was not required. As such, these results largely reflect the short- and intermediate-term impacts, because long-term outcomes are still being assessed by lead agencies. Despite this, initial impacts strongly suggest that with continued advocacy activity, stakeholders are making notable progress toward seeing long-term impacts. Another limitation is the lack of data collected from patients who participated in the projects. The scale of our evaluation limited us to surveying lead agencies with which we worked directly. In addition, given the complexities of policy and system change in particular, the framework should be viewed as a mechanism to contribute to long-term outcomes, as opposed to being the sole factor to which long-term outcomes are attributed.

In conclusion, given our results, the work by Durstine and Leitman,^25^ and illness narrative studies like that by Green,^36^ we suggest that there is an opportunity to further study the effects of patient storytelling on personal and collective empowerment and its influence on disclosure of diagnosis, contributing to reduction of stigma. On the basis of limited qualitative data collected from participants in the Japanese training, it is suggested that the framework benefitted patients by providing a meaningful way to engage in the fight against cancer, connect with other patients in a supportive setting, and to heal and find meaning in the cancer journey.^36^ We suggest that future research explore the impact of patient storytelling globally as a means of social support and psychosocial healing as well as its impact on influencing others to engage in grassroots advocacy.

On the basis of these results, we believe that the IPAF is globally relevant and could be adapted and implemented in other countries to amplify patient voices in the policymaking process, increase grassroots mobilization, improve health systems through addressing patient needs, and ultimately support countries in meeting NCD and cancer control targets. Further research is also needed on the long-term impacts of cancer patient advocacy; it would be particularly beneficial to see other countries implement the framework and track outcomes longitudinally. Ultimately, people will benefit if health systems globally can evolve toward a place of sustained and meaningful patient participation and engagement.
